# Increase in circulating sphingosine-1-phosphate and decrease in ceramide levels in psoriatic patients

**DOI:** 10.1007/s00403-016-1709-9

**Published:** 2016-12-17

**Authors:** Hanna Myśliwiec, Anna Baran, Ewa Harasim-Symbor, Barbara Choromańska, Piotr Myśliwiec, Anna Justyna Milewska, Adrian Chabowski, Iwona Flisiak

**Affiliations:** 1 Department of Dermatology and Venereology, Medical University of Bialystok, Żurawia Str. 14, 15-540 Bialystok, Poland; 2 Department of Physiology, Medical University of Bialystok, Bialystok, Poland; 3 I Department of General and Endocrinological Surgery, Medical University of Bialystok, Bialystok, Poland; 4 Department of Statistics and Medical Informatics, Medical University of Bialystok, Bialystok, Poland

**Keywords:** Psoriasis, Psoriatic arthritis, Ceramide, Sphingosine-1-phosphate, Sphingolipids

## Abstract

Psoriasis is characterized by hyperproliferation, deregulated differentiation and impaired apoptosis of keratinocytes. Mechanisms of lipid profile disturbances and metabolic syndrome in the psoriatic patients are still not fully understood. Sphingolipids, namely ceramides (CER) and sphingosine-1-phosphate (S1P) are signal molecules which can regulate cell growth, apoptosis and immune reactions. The aim of the study was to evaluate circulating CER and S1P levels in plaque-type psoriasis and their associations with the disease activity, inflammatory or metabolic markers and the presence of psoriatic comorbidities. Eighty-five patients with exacerbated plaque-type psoriasis and thirty-two healthy controls were enrolled. Serum CER and S1P concentrations before the treatment were examined. General patient characteristics included: PASI (Psoriasis Area and Severity Index), BMI (Body Mass Index), inflammatory and biochemical markers, lipid profile and presence of psoriatic comorbidities. Total serum concentration of CER was significantly decreased (*p* = 0.02) and concomitantly S1P levels significantly increased (*p* = 0.002) in psoriatic patients compared to the healthy control group. Among patients with psoriasis no significant correlations with the disease activity and inflammation markers were observed and only patients with psoriatic arthritis had significantly higher CER total concentration. Serum sphingolipid disturbances in psoriatic patients were observed. Decreased total CER and increased S1P serum levels may reflect their epidermal altered composition and metabolism. Patients with psoriatic arthritis have higher CER levels than psoriasis with skin involvement only. It might provide additional predictive value for psoriatic arthritis and may convey higher risk of metabolic and cardiovascular disease development in this group of patients.

## Introduction

Psoriasis is an immune-mediated chronic inflammatory disease which affects approximately 1–11% of the world’s population [37]. Typical skin lesions are characterized by hyperproliferation, deregulated differentiation of epidermal keratinocytes and infiltration of immune cells into the skin [29]. In psoriasis, decreased spontaneous keratinocytes apoptosis in lesional skin was found [24]. Additionally, keratinocytes in psoriatic plaques are characterized by resistance to apoptosis compared with normal keratinocytes [42].

Several recent studies have shown that psoriasis is not only a skin disease but is also connected to many systemic disturbances [14]. Psoriasis is associated with metabolic syndrome, which is defined as a constellation of distinct clinical entities: insulin resistance, obesity, hyperlipidemia and hypertension. Their progression leads to atherosclerotic vascular disease and type 2 diabetes. It has been established that the release of inflammatory molecules and cytokines may play an important role in this association [9].

Lipid profile disturbances in the psoriatic patients were reported previously. Serum triglycerides, cholesterol and LDL had significantly higher concentration in psoriatic patients when compared to healthy controls [1, 13], while the high-density lipoprotein cholesterol was significantly decreased [13].

Sphingolipids have structural functions in the human skin. They are important for the development of epidermal barrier. In the recent years it became clear that sphingolipids are not only structural components of the skin but also their derivatives are signal molecules that regulate biological functions of keratinocytes and immune cells of the skin [5]. Among most biologically active sphingolipids are ceramides (CER) and sphingosine-1-phosphate (S1P). They have different signaling roles. CER are involved in apoptosis, cell cycle arrest, inflammation and stress responses [5]. On the contrary, S1P is a signaling molecule, taking part in the regulation of many different cellular functions including cell growth, differentiation, proliferation and migration. Binding of S1P to its cell surface receptors initiates angiogenesis. Interestingly, the effect of S1P on epidermal cells significantly differs from most other cells, as it inhibits keratinocytes’ proliferation and induces their differentiation and migration [18]. S1P antagonizes CER-mediated apoptosis in healthy skin. The studies performed on psoriatic skin revealed decrease in total amount of CER comparing lesional to non-lesional epidermis [25] and increase in sphingosine, a well-established precursor of S1P [35].

Disturbed intracellular sphingolipids metabolism has been recently implicated also in the development of several diseases such as obesity [3], type 2 diabetes [28], artherosclerosis and cardiovascular diseases [26] and arthritis [20]. Importantly, cellular changes were also reflected by modified serum sphingolipid levels as recently demonstrated by Yu et al. showing that CER levels are increased in chronic heart failure and associated with the severity of clinical symptoms [43]. Other research confirmed also that serum CER is associated with atherogenic lipid profiles and insulin resistance in obesity [30].

Multiple studies have examined the dysregulation of sphingolipids metabolism in psoriatic skin, but there are currently limited data on the role of circulating levels in psoriasis.

The aim of the present study was to evaluate selected circulating CER and S1P levels in exacerbated plaque-type psoriasis and their correlation with the clinical disease severity, inflammatory markers, serum lipid profile, vitamin D concentration and possible involvement in psoriatic comorbidities: psoriatic arthritis, diabetes mellitus type 2, hypertension and obesity.

## Materials and methods

Eighty-five patients (28 females and 57 males) with active plaque-type psoriasis, at median age 53 (19–79 years) and 32 sex- and age-matched healthy controls were included in the study. The severity of psoriasis was estimated using Psoriasis Area and Severity Index (PASI) [39]. Patients were divided into three groups with mild (PASI < 10), moderate (PASI between 10 and 20), and severe (PASI > 20) psoriasis.

Body mass index (BMI) was calculated based on self-reported weight and height. Overweight was defined as BMI ≥25 kg/m^2^ and obesity as BMI ≥30 kg/m^2^. The history of hypertension and diabetes as well as results of the laboratory tests were collected from hospital records of the patients.

All patients and controls gave their written informed consent before the enrollment. The study protocol was approved by local bioethical committee.

Peripheral blood samples were taken before starting the treatment from patients and from the control group. After centrifugation, the serum was stored at −80 °C until analyses.

Briefly, the serum samples were mixed with a solution composed of 25 mM HCl and 1 M NaCl and acidified with methanol. Internal standards of C17–sphingosine and C17–sphingosine 1-phosphate (Avanti Polar Lipids, Alabaster, AL, USA) were added. Lipids were extracted by means of chloroform, 1 M NaCl and 3 N NaOH. The aqueous phase containing S1P was transferred to a fresh tube and the compound was dephosphorylated with the use of alkaline phosphatase (bovine intestinal mucosa, Fluka). Free sphingosine were converted to their *O*-phthalaldehyde derivatives and analyzed by means of high-performance liquid chromatography (HPLC) system equipped with fluorescence detector and C18 reversed-phase column (Varian Inc., OmniSpher 5, 4.6 × 150 mm).

To quantify CER, a small volume of the chloroform phase containing lipids was transferred to a tube containing *N*-palmitoyl-d-erythro-sphingosine (C17 base) as an internal standard. The lipid fractions were separated by thin-layer chromatography silica plates (Kieselgel 60, 0.22 mm, Merck, Darmstadt, Germany) with a heptane:isopropyl ether:acetic acid (60:40:3, vol/vol/vol) resolving solution. Lipid bands were visualized by spraying with a 0.2% solution of 3′7′-dichlorofluorescin in methanol and identified under ultraviolet light using standards on the plates. The gel bands were scraped off the plate, transferred into screw tubes and transmethylated with BF3/methanol. The fatty acid methyl esters (FAMEs) were dissolved in hexane and analyzed by gas–liquid chromatography. A Hewlett-Packard 5890 Series II gas chromatograph with Varian CP-SIL capillary column (50 m 0.25 mm internal diameter) and flame-ionization detector (Agilent Technologies, Santa Clara, CA) was used. Injector and detector temperatures were set at 250 °C. The oven temperature was increased linearly from 160 to 225 °C at a rate of 5 °C/min. According to the retention times of standards, the individual long-chain fatty acids were quantified. Total content of CER was estimated as the sum of the particular fatty acid species of the assessed fraction and it was expressed in nanomoles per milliliter of the serum.

Data were presented as median and quartiles (first and third quartile) and percentage when appropriate. After analysis of distribution, the statistical analysis was performed using Kruskal–Wallis and Mann–Whitney tests. The level *p* < 0.05 was regarded as significant. The correlations between the variables were calculated using non-parametric Spearman’s test.

## Results

Eighty-five patients (28 females and 57 males) aged 19–53 (mean 49.7 ± 14.4 years) with exacerbated plaque-type psoriasis and 32 age- and sex-matched healthy controls were included in the study. The duration of psoriasis ranged from 1 to 58 months (mean 18.5 ± 14.4 months). Mean score of BMI was 28.5 ± 6.3 and PASI score 11.4 ± 8.7. Fifty persons (58.8%) had mild psoriasis (PASI < 10), 22 (25.8%) had moderate psoriasis (PASI between 10 and 20) and 13 (15.3%) severe (PASI > 20). Patients were evaluated according to present psoriatic comorbidities. Fourteen patients (16.5%) were diagnosed with psoriatic arthritis, 13 (15.3%) with type 2 diabetes, 29 (34.1%) suffered from hypertension, 31 (36.5%) patients were overweight (BMI > 25) and 25 (29.4%) had obesity (BMI > 30), 16 (18.8%) patients had hypercholesterolemia (>200 mg/dl) and 15 (17.6%) hypertriglyceridaemia (>160 mg/dl) and 9 (10.6%) patients history of cardiovascular diseases. The selected demographic, clinical and laboratory data are summarized in Table 1.

**Table 1:** Clinical and laboratory characteristics of psoriatic patients

Patients (*n* = 85)	Median (Q_1_; Q_3_)
Age	53.0 (41.0; 59.0)
Gender: F/M	28 (33%)/57 (67%)
BMI	27.18 (23.89; 31.60)
Psoriasis duration (months)	17.0 (6.0; 29.0)
PASI	9.00 (5.50; 14.7)
C-reactive protein (mg/l)	2.55 (1.15; 5.85)
White blood cells (×10^3^/ml)	6.93 (5.92; 8.14)
Platelets (×10^3^/ml)	215 (190; 257)
Serum glucose (mg/dl)	88 (77; 98)
Cholesterol (mg/dl)	177 (156; 198)
Triglyceride (mg/dl)	109 (79; 149)
Vitamin D (ng/ml)	27.18 (11.53; 21.92)

Data shown as median and quartiles (Q_1_—first quartile; Q_3_—third quartile)

**Fig. 1:**
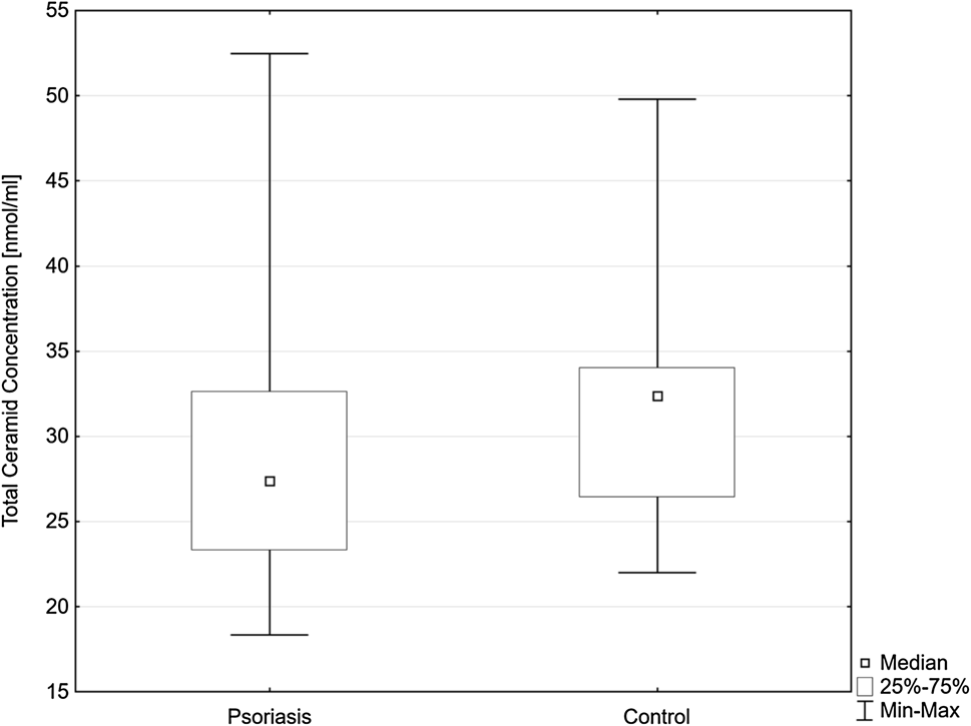
Total ceramide concentrations in serum of the psoriatic patients (Psoriasis) and the control group (Control). Data shown as median (Q_1_, Q_3_), significant differences between the groups *p* = 0.02*

Total serum concentration of CER was significantly lower in psoriatic patients than in the control group (Fig. 1). Selected CER concentration in comparison to the control group is shown in the Table 2. Serum total CER concentration did not correlate with the psoriasis severity measured by PASI, time of the duration of the disease nor the investigated laboratory results: C-reactive protein, white blood cell count, platelet count, fasting glucose, vitamin D concentration. Patients with concomitant psoriatic arthritis had significantly higher total CER concentration and concentration of certain CER (Table 3). FA-C22 ceramide concentration correlated with total cholesterol concentration (*R* = 0.399), triglyceride concentration (*R* = 0.375) and was significantly higher in psoriatic patients with obesity (*p* = 0.014). Total CER concentration in serum of psoriatic patients suffering diabetes type 2 or hypertension did not differ from those in psoriatic patient without these disorders.

**Table 2:** Differences between serum ceramides (CER) (nmol/ml) and sphingosine-1-phosphate (pmol/ml) concentrations in psoriatic patients and the control group

Ceramide (CER)	Psoriatic patients Median (Q_1_, Q_3_)	Controls Median (Q_1_, Q_3_)	*p* value
CER myristic (C14:0)	1.42 (1.06; 2.94)	3.59 (1.22; 5.02)	0.002**
CER palmitic (C16:0)	8.01 (6.47; 9.55)	7.90 (7.04; 9.06)	0.76
CER palmitoleic (C16:1)	0.66 (0.56; 1.09)	0.86 (0.70; 1.08)	0.05*
CER stearic (C18:0)	6.58 (5.55; 7.86)	5.63 (4.67; 7.52)	0.05
CER oleic (C18:1)	1.82 (1.74; 2,12)	2.07 (1.85; 2.42)	0.005**
CER linoleic (C18:2)	0.05 (0.00; 0.34)	0.23 (0.04; 0.46)	0.03*
CER arachidic (C20:0)	0.41 (0.37; 0.48)	0.46 (0.41; 0.55)	0.04*
CER linolenic (C18:3)	0.18 (0.16; 0.22)	0.21 (0.18; 0.25)	0.007**
CER behenic (C22:0)	1.20 (1.10; 1.43)	1.28 (1.12; 1.43)	0.57
CER arachidonic (C20:4)	0.32 (0.29; 0.39)	0.42 (0.33; 0.53)	0.002**
CER lignoceric (C24:0)	2.97 (2.47; 3.44)	3.48 (3.15; 3.94)	0.0003***
CER eicosapentaenoic (C20:5)	0.00 (0.00; 0.00)	0.56 (0.00; 0.74)	0.0001***
CER nervinic (C24:1)	2.05 (1.91; 2.26)	2.04 (1.88; 2.23)	0.71
CER docosahexaenoic (C22:6)	0.50 (0.00; 0.55)	0.56 (0.54; 0.60)	0.0001***

Data shown as median and quartiles (*Q*_*1*_ first quartile, *Q*_*3*_ third quartile). Significant differences between the groups are shown as: * −*p* < 0.05, ** −*p* < 0.01, ***−*p* < 0.001

**Table 3:** Serum concentrations of ceramides (CER) (nmol/ml) and sphingosine-1-phosphate (pmol/ml) in psoriasis and psoriatic arthritis

Sphingolipids	Psoriasis Median (Q_1_, Q_3_)	Psoriatic arthritis Median (Q_1_, Q_3_)	*p* value
CER myristic (C14:0)	1.31 (1.01; 1.85)	2.94 (1.78; 4.02)	0.003**
CER palmitic (C16:0)	7.44 (6.06; 9.52)	8.79 (8.39; 10.16)	0.01*
CER palmitoleic (C16:1)	0.66 (0.54; 1.12)	0.92 (0.59; 1.05)	0.51
CER stearic (C18:0)	6.23 (5.38; 6.76)	7.08 (6.72; 9.01)	0.03*
CER oleic (C18:1)	1.82 (1.74; 2.08)	1.99 (1.74; 2.17)	0.52
CER linoleic (C18:2)	0.05 (0.00; 0.29)	0.016 (0,00; 0.37)	0.95
CER arachidic (C20:0)	0.41 (0.37; 0.47)	0.45 (0.39; 0.60)	0.27
CER linolenic (C18:3)	0.18 (0.16; 0.22)	0.19 (0.17; 0.21)	0.87
CER behenic (C22:0)	1.20 (1.11; 1.42)	1.19 (1.09; 1.47)	0.98
CER arachidonic (C20:4)	0.31 (0.29; 0.39)	0.35 (0.32; 0.38)	0.28
CER lignoceric (C24:0)	2.93 (2.48; 3.31)	3.11 (2.31; 3.65)	0.62
CER eicosapentaenoic (C20:5)	0.00 (0.00; 0.00)	0.00 (0.00; 0.55)	0.02*
CER nervinic (C24:1)	2.04 (1.91; 2.29)	2.10 (1.90; 2.24)	0.82
CER docosahexaenoic (C22:6)	0.50 (0.00; 0.55)	0.00 (0.00; 0.54)	0.38
CER total	26.12 (22.03; 32.22)	31.72 (29.26; 35.69)	0.003**
Sphingosine-1-phosphate	510.4 (452.7; 558.1)	473.9 (388.8; 570.9)	0.35

Significant differences between the groups are shown as: * −*p *< 0.05, ** −*p* < 0.01

**Fig. 2:**
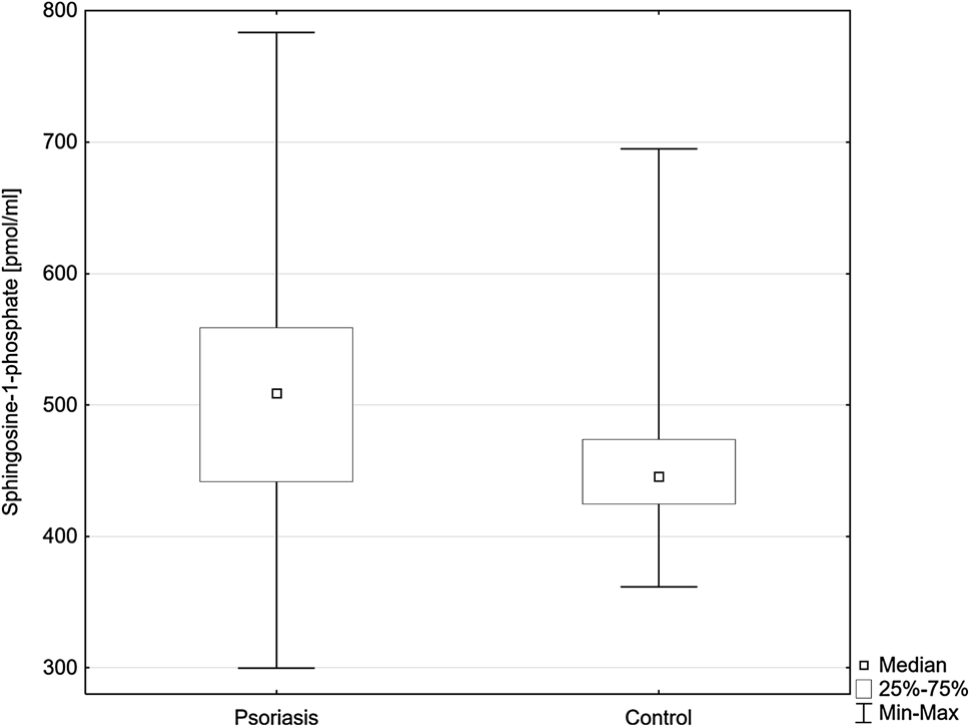
Sphingosine-1-phosphate concentrations in serum of the psoriatic patients (Psoriasis) and the control group (Control). Data shown as median (Q_1_, Q_3_). Significant differences in the control group are shown as: *p* = 0.002**

S1P serum concentration was significantly higher in psoriatic patients than in the control group (Fig. 2), but was not related to psoriasis severity, nor duration of the disease. We have not found any significant correlations between S1P concentration and laboratory results, BMI nor with the presence of comorbidities.

## Discussion

We examined circulating sphingolipid levels in psoriatic patients with respect to clinical and laboratory data. In the present study, we have demonstrated significantly lower serum CER levels in the patients with psoriasis compared with the healthy subjects. There are limited data in the recent literature concerning circulated CER in the psoriatic patients. We may speculate that reduced circulating levels of CER, obtained in our study, may reflect their lower levels in the psoriatic skin as diminished CER content in the lesional epidermis in the psoriasis was recently observed [25]. Importantly, psoriatic skin lesions expressing reduced levels of CER lead to an anti-apoptotic and pro-proliferative epidermal environment, and subsequently to overproliferation of keratinocytes and the development of lesions [25]. Other research confirmed also reduction of CER synthesis (ranging from 4.3 to 78.8%) in the lesional psoriatic epidermis compared to unlesional epidermis [10]. Interestingly, the authors demonstrated highly significant, positive correlation between the percentage reduction of CER synthesis and PASI score in mild and moderate psoriasis. Additionally, a very recent study revealed also alterations in the CER fatty acid profile in the stratum corneum of psoriatic patients. Proportion of CER with long-chain fatty acids was significantly lower in psoriasis patients than in controls [40]. In our study, we have evaluated only CER with long-chain fatty acids and we do not compare the proportions with other authors.

There are numerous studies confirming depletion of CER in psoriatic epidermis [10, 40]. To our knowledge, however, there are no studies evaluating the correlation between CER content in the skin and the circulating level of CER. CER levels depend on constant balance between their production and degradation. Production of CER in psoriasis is probably impaired because of reduced CER synthase activity [40], decreased sphingomyelinase activity—another important enzyme involved in CER synthesis and decreased level of prosaponin—a saponin precursor, which is a non-enzymatic cofactor of hydrolysis of sphingolipids [2].

But there are also conflicting findings. Increased levels of CER were observed by Checa et al. both in serum and in lesional skin relative to non-lesional and control skin, but only in severe psoriasis [8]. Their results indicate that although epidermal lipid synthesis is largely independent of systemic lipids, probably these two compartments are interrelated, especially in a pathologic condition like psoriasis. In this context, our results stay in line with the majority of reports that point out depletion of CER in psoriasis [10, 40]. Interestingly, we have demonstrated, to our knowledge for the first time, increased levels of circulating CER in patients with psoriatic arthritis compared to psoriasis without arthritis. The group with joint disease was considered as the group of patients with more severe disease. Based on our results, circulating CER did not correlate with other systemic inflammatory markers or PASI. However, maybe the level of circulating CER is more sensitive than C-reactive protein or white blood cells and will help to distinguish the group with joint involvement or even predict the join involvement. A hypothesis should be certainly verified by further prospective studies.

Sphingolipids and CER among them are components of synovial fluid and can take part in arthritis pathology. In rheumatoid arthritis, an overgrowth of synovial cells was described, which results in joint destruction. It is probably due, at least in part, to impaired balance between cell proliferation and apoptosis. CER may be highly involved in this process [33] based on its proapoptotic potential, as shown in several studies conducted on synovial cells from patients with rheumatoid arthritis [34]. Altered composition of synovial fluid was reported in osteoarthritis and rheumatoid arthritis. Kosinska et al. [20] found elevated total CER concentration in synovial fluid of these inflammatory joint diseases in comparison with control healthy synovial fluid. There are virtually no such data concerning psoriatic arthritis; however, findings of our study suggest the possible role of CER also in psoriatic arthritis. The present knowledge gap regarding this relationship needs to be clarified in the future.

It is estimated that about 30% of psoriatic patients will develop psoriatic arthritis [15]. A retrospective analysis conducted on a very large population in the United Kingdom, identified patients with psoriatic arthritis as having significantly higher prevalence of type 2 diabetes mellitus, hypertension, rheumatoid arthritis and ankylosing spondylitis when compared to psoriatic patients without arthritis [12]. The reason for this finding remains unclear. Higher rates for comorbidities could be an effect of chronic inflammation, production of proinflammatory cytokines and consecutive endothelial damage, as it was suggested in psoriasis [31]. CER levels have been reported to increase in chronic heart failure and to be associated with the severity of clinical symptoms [43]. Also high S1P serum levels have been reported as a predictive factor of obstructive artery disease [11]. Concordant with Checa et al. [8], we propose that the association of CER and cardiac involvement is most likely restricted to patients with more severe disease. Increased CER are a potential link between severe psoriasis and cardiovascular disease. CER derived from sphingomyelins have been reported to be implicated in atherosclerotic plaque formation [7]. CER are regarded as important second messengers in the atherosclerotic processes. Sphingomyelin, which is transported into the arterial wall by atherogenic lipoproteins, is transformed by arterial wall sphingomyelinase into CER, promoting in turn lipoprotein aggregation [4].

There are some diseases characterized by reduced CER level in the circulation. Significant decrease in levels of long and very long chain CER were observed in patients with severe cirrhosis. Additionally, an association between low serum concentrations of FA-C24 ceramide and hepatic decompensation as well as poor overall survival was observed [16]. The same CER was diminished in the serum of HCV patients [17]. Another study revealed that low levels of very long chain CER were associated with the development of macroalbuminuria in patients with type 1 diabetes [19]. It seems that very long chain CER may have protective role in hepatic and renal homeostasis and their significant decrease may be predictive of organ failure. The authors suggest that the decrease in serum FA-C24 ceramide probably shifts the balance between proliferation and apoptosis in favor of a proapoptotic state, and finally accelerate clinical decompensation and mortality in cirrhosis [16]. The underlying mechanisms need further investigations.

Serum CER levels were suggested to be associated with several metabolic disorders such as obesity and diabetes so we have examined the possible association of CER with metabolic disorders in the course of psoriasis. In our settings, there were no statistical differences in the circulating CER levels between patients with hypertension (*N* = 29), type 2 diabetes (*N* = 13), or cardiovascular diseases (*N* = 9) and those without comorbidities. Nevertheless, our group of psoriatic patients was limited (*N* = 85) and there were only small group of patients with above-mentioned comorbidities. It would be useful to conduct a larger, ideally prospective study.

In our study, we found that circulating serum S1P is elevated in psoriasis compared to healthy control group. S1P is the breakdown product of CER, and in the human-cultured keratinocytes S1P inhibited proliferation and promoted differentiation [18]. S1P can not only affect epidermal proliferation rate balance but can also modulate immunological response by regulation of circulation of T lymphocytes between lymph, plasma and tissue. In the recent years, it has been found that stimulation of S1P receptor 1 caused lymphocytes T migration out of lymphatic tissue [6]. Circulating S1P plays a significant physiological role. Within the plasma, most S1P is bound to protein carriers, such as HDL (~60%) and albumin (~30%), with lesser amounts bound to VLDL and LDL [36]. It was found to be the key regulator of lymphocyte trafficking, endothelial barrier function and vascular tone. In pathology, S1P metabolism is associated with inflammatory and autoimmune diseases: rheumatoid arthritis [23], multiple sclerosis [41] and cardiovascular diseases [27].

CER can be transformed to sphingosine further to S1P, which might at least in part explain the decrease in CER and increase in S1P found in our patients. Even though there are no data available on increased ceramidase activity in psoriatic patients, Moon et al. [35] described a highly significant positive correlation between the % change of ceramidase activity in the lesional skin of psoriatic patients and PASI score.

The majority of circulating S1P comes from erythrocytes, leukocytes, vascular endothelial cells and hepatocytes [21]. However, there is a possibility that other kinds of cells directly affected in psoriasis can be involved. To our knowledge there is no direct evidence of an association between the amount of circulating S1P and the activity of the disease. Indirectly, we can deliberate that levels of S1P are controlled by the enzymes responsible for its synthesis and degradation. Mechtcheriakova et al. [32] detected significantly increased mRNA expression of S1P phosphatase 2, the enzyme hydrolyzing S1P back to sphingosine, in psoriatic lesions compared to non-lesional skin. Another study confirms higher levels of sphingosine in the lesional epidermis [35].

Noteworthy, our results stay in line with few previous studies, reporting also [8] elevated levels of circulating S1P in severe psoriasis compared to patients with mild disease and with the healthy individuals. Nevertheless, it has recently been revealed that plasma S1P levels in obesity are elevated when compared to lean controls [22]. The authors also observed significant correlation with clinical indices of metabolic syndrome such as waist circumference, body fat percentage, fasting plasma insulin, total and LDL cholesterol. In another study, serum S1P levels have been shown to have predictive value of both the occurrence and severity of coronary stenosis [11]. Authors proposed the novel role of sphingolipids in the pathogenesis of obesity-mediated cardiovascular and metabolic disease [38]. In our psoriatic group, there were only 13 patients with type 2 diabetes, 29 with hypertension, 31 patients were overweight and 25 had obesity. This small numbers of patients may not be sufficient to observe significant differences in S1P concentration.

In our study, we identified potentially important circulating S1P differences between psoriasis and healthy controls. Further studies on larger sample group are needed to confirm our results.

In conclusion, we observed significantly lower serum CER concentration and higher S1P concentrations in psoriatic patients compared to the control group. Sphingolipid serum disturbances may reflect their epidermal altered composition and metabolism. A pathogenic link may exist between the certain species of sphingolipids and psoriatic pathophysiology. Moreover, we revealed higher serum CER levels in psoriatic arthritis than in the psoriasis with skin lesions only. It might provide additional predictive value for psoriatic arthritis and may convey higher risk of metabolic and cardiovascular diseases development in this group of patients.
